# Research on Acoustic Field Correction Vector-Coherent Total Focusing Imaging Method Based on Coarse-Grained Elastic Anisotropic Material Properties

**DOI:** 10.3390/s25154550

**Published:** 2025-07-23

**Authors:** Tianwei Zhao, Ziyu Liu, Donghui Zhang, Junlong Wang, Guowen Peng

**Affiliations:** 1 School of Resources, Environment and Safety Engineering, University of South China, Hengyang 421001, China; c23ztw@163.com; 2 China Nuclear Industry 23 Construction Co., Ltd., Beijing 101300, China; 3 NDT&E Laboratory, Dalian University of Technology, Dalian 116024, China; liuzyu0818@163.com

**Keywords:** ultrasonic phased array, TFM, coarse-grained material, sound field correction, phase coherent imaging

## Abstract

This study aims to address the challenges posed by uneven energy amplitude and a low signal-to-noise ratio (SNR) in the total focus imaging of coarse-crystalline elastic anisotropic materials. A novel method for acoustic field correction vector-coherent total focus imaging, based on the materials’ properties, is proposed. To demonstrate the effectiveness of this method, a test specimen, an austenitic stainless steel nozzle weld, was employed. Seven side-drilled hole defects located at varying positions and depths, each with a diameter of 2 mm, were examined. An ultrasound simulation model was developed based on material backscatter diffraction results, and the scattering attenuation compensation factor was optimized. The acoustic field correction function was derived by combining acoustic field directivity with diffusion attenuation compensation. The phase coherence weighting coefficients were calculated, followed by image reconstruction. The results show that the proposed method significantly improves imaging amplitude uniformity and reduces the structural noise caused by the coarse crystal structure of austenitic stainless steel. Compared to conventional total focus imaging, the detection SNR of the seven defects increased by 2.34 dB to 10.95 dB. Additionally, the defect localization error was reduced from 0.1 mm to 0.05 mm, with a range of 0.70 mm to 0.88 mm.

## 1. Introduction

An austenitic stainless nozzle weld is the weld seam of the protruding pipe seat in the nuclear auxiliary system of a nuclear power plant that connects a small branch to the main pipeline [1]. In the event of a leak during operation, the system’s safe and stable functioning is significantly compromised, making the application of appropriate non-destructive testing methods essential for ensuring quality control [2,3,4,5]. The Total Focusing Method (TFM) [6] has proven advantageous in the inspection of nozzle welds [7,8,9,10]. This method calculates the time-delay superposition of ultrasonic signals to achieve virtual focusing at every point in the imaging space. Compared to phased array scanning imaging, TFM offers enhanced spatial uniformity and a broader single-scan coverage area, earning its reputation as the “gold standard” in phased array ultrasonic testing.

TFM assumes that the amplitude weights of each imaging point remain constant during the time-delay stacking process. In isotropic homogeneous materials, the directionality of the acoustic field and the diffusion of ultrasound waves can lead to uneven energy distribution across different locations, causing image distortion, artifacts, and reduced detection accuracy [11]. Current research on optimizing amplitude stacking weights for homogeneous materials primarily focuses on three approaches: First, simulation or experimental methods are employed to optimize detection parameters and reduce amplitude non-uniformity within the imaging space [12,13]. Second, combining sound field analysis with distance amplitude correction methods [14], distance gain size curves [15], and other quantitative assessment techniques is essential. Third, establishing a sound field correction function based on sound field directionality is necessary to compensate for energy variations in the imaging region, offering good adaptability and strong interpretability [16]. Among these factors, the selection of the acoustic field directivity function and compensation factors is the key determinant of the compensation’s effectiveness. Zhou et al. [17] developed a diffusion attenuation model for a two-layer medium based on the acoustic field directivity function. They calculated the ultrasonic energy attenuation coefficient and successfully detected a 1.5 mm diameter transverse through-hole in a B-type phased array standard test block at depths of 10 mm and 12 mm, which would have been undetectable without calibration, but were identified using this method. Zhu et al. [18] analyzed the phased array ultrasonic testing of rails and introduced acoustic path compensation, integrating it with sparse array TFM. In scenarios with a sparsity rate of less than 75%, the imaging signal-to-noise ratio (SNR) closely matched that of a full-array TFM image. The optimization of amplitude superposition weights in austenitic stainless steel welds, which are coarse-grained and exhibit elastic anisotropy, is complicated by several factors that reduce the quality of TFM correction imaging. Firstly, the coarse-grained structure of the weld causes ultrasonic wave scattering attenuation. Conventional acoustic field correction functions fail to effectively compensate for energy non-uniformity, making it challenging to accurately measure scattering attenuation. Secondly, the weld’s elastic anisotropy introduces structural noise into the image background, which negatively impacts the SNR and defect localization accuracy [19,20,21,22,23]. Acoustic field correction methods cannot distinguish between defects and structural noise, leading to indiscriminate compensation of energy amplitude across the imaging region, which ultimately reduces SNR in certain areas. Phase-coherent imaging (PCI), by considering the phase information of ultrasonic signals, adapts to identify both defects and background noise, thereby improving SNR [24,25]. Among these methods, vector-coherent imaging has demonstrated superior imaging efficiency, avoiding local image distortion—a limitation of signal-coherent imaging. This characteristic provides vector-coherent imaging with a distinct advantage in terms of noise reduction for TFM imaging [26,27].

This study investigates an austenitic stainless steel weld test block. Based on the crystal orientation and microstructure characterization results retrieved by Electron Backscatter Diffraction (EBSD) technology, the attenuation of ultrasonic waves in different regions is analyzed, and the scattering attenuation compensation factors in the base metal and weld regions are optimized. It also establishes an acoustic field correction function based on material properties, integrates phase information, and extracts phase coherence from the ultrasonic signals. Calculating the phase coherence weighting coefficients is essential for effective energy compensation and defect reconstruction imaging. This process enhances spatial averaging, suppresses background noise, and enables material property-based acoustic field correction vector-coherent full-field imaging. The effectiveness of this approach is validated through empirical evidence from experimental studies.

## 2. Principle

### 2.1. TFM Imaging

**Figure 1:**
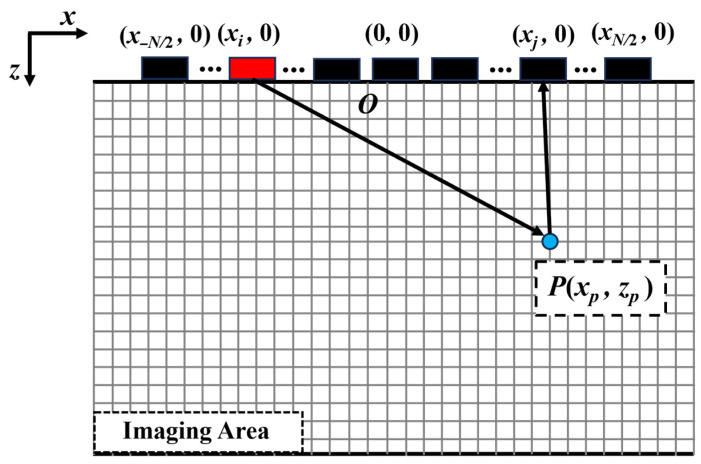
Schematic diagram illustrating principle of TFM imaging.

TFM relies on Full Matrix Capture (FMC) data, where each array element is sequentially excited to emit ultrasound pulses, and the echo signals are received through all array elements. The principle is illustrated in Figure 1. In the imaging region, pixel sizes are defined, the grid is segmented, and a Cartesian coordinate system is established, with the *x*-axis parallel to the array arrangement direction and the *z*-axis perpendicular to the surface of the sample under test. Assuming there are Ne array elements, for any focal point *P* (*x_p_*, *z_p_*) within the imaging region, the delay rule is determined based on the acoustic path length from each array element to the focal point *P*, and the forward signal amplitude is processed by summation. The total response amplitude of all signals passing through point *P* is calculated as follows:

(1) $$I_{\mathrm{TFM}}\left(x_{p},z_{p}\right)=\left|H\left(\sum_{i=1}^{N_{e}}\sum_{j=1}^{N_{e}}S_{ij}\left(t_{ij}\left(x_{p},z_{p}\right)\right)\right)\right|$$

where *H* denotes the Hilbert transform, *S_ij_* (*t*) represents the amplitude information at the focal point *P* in the ultrasonic echo signal emitted by element *i* and received by element *j*, and *t_ij_* (*x_p_*, *z_p_*) denotes the time taken for the ultrasonic wave to travel through point *P*:

(2) $$t_{ij}\left(x_{p},z_{p}\right)=\frac{\sqrt{{\left(x_{i}-x_{p}\right)}^{2}+z_{p}^{2}}+\sqrt{{\left(x_{j}-x_{p}\right)}^{2}+z_{p}^{2}}}{c_{L}}$$

where *c_L_* is the longitudinal wave velocity of the test block.

Conversely, by acquiring the total response amplitude at different focal points, the TFM image of the corresponding region can be reconstructed.

### 2.2. Sound Field Correction and Compensation

Austenitic stainless steel welds are imaged using the TFM imaging technique that employs time-delay superposition to achieve virtual focusing at each pixel within the imaging space. However, three issues arise during the time-delay superposition process, which negatively impact the quality of the TFM image. First, the directionality of the acoustic field leads to inconsistent acoustic energy at different angles, resulting in a lack of uniformity in the TFM image. Second, variations in the acoustic path length cause differences in the diffusion attenuation of ultrasonic waves, leading to inconsistent amplitude energy at different imaging positions. Third, the coarse grain structure and anisotropy of the austenitic stainless steel weld cause varying scattering attenuation of ultrasonic signals in different regions, resulting in uneven image amplitude. Therefore, it is essential to separately compensate and correct each of these three factors to minimize the interference caused by image non-uniformity.

#### 2.2.1. Sound Field Directivity Function and Diffusion Attenuation Compensation

In two-dimensional or three-dimensional space, the sound field directivity function describes the relationship between the sound field energy of each array element and the propagation direction of the ultrasound beam. By calculating the distribution of sound field energy within the TFM imaging space, compensation and correction can be applied to reduce interference caused by the non-uniformity of the imaging amplitude distribution.

**Figure 2:**
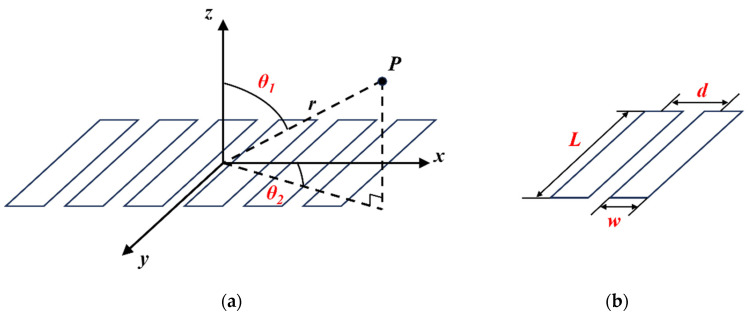
Array element structure and associated parameters: (**a**) deflection angle; (**b**) array element size parameters.

The configuration of the phased array elements is illustrated in Figure 2. The length of a single element is denoted by *l*, the width by *w*, the center-to-center distance between two adjacent elements by *d*, and the distance from a point *P* in the far field to the center of the element by *r_iP_*. In the context of two-dimensional imaging, the acoustic field directivity function can be expressed as follows [18]:

(3) $$D_{i}\left(\theta_{1}\right)=\sin\left(\frac{\pi d\sin\theta_{1}}{\lambda }\right)$$

where *θ*_1_ represents the angles between the projections of r onto the xoz planes, respectively, and the z-axis. The wavelength of the ultrasonic wave, denoted as *λ*, is a crucial variable in this equation.

To understand the characteristics of array element transmission and reception in FMC, it is essential to consider both the transmission and reception sound fields when implementing sound field directivity compensation. Specifically, when an array element transmits to point *P* in the far field and is received by an array element designated as “*j*,” the directivity compensation factor of the sound field can be expressed as follows:

(4) $$D_{ij}\left(x_{P},z_{P}\right)=D_{ij}\left(\theta_{1}\right)=\sin\left(\frac{\pi d\sin\theta_{i1}}{\lambda }\right)\cdot \sin\left(\frac{\pi d\sin\theta_{j1}}{\lambda }\right).$$

Furthermore, due to the varying diffusion attenuation caused by different sound paths of ultrasonic waves, a diffusion attenuation compensation factor is introduced. According to Green’s function, in two-dimensional space, the diffusion attenuation at point *r*, away from the point source, is equal to $\sqrt{\frac{1}{4\pi r}}$. When element *i* transmits to point *P* in the far field and is received by element *j*, the diffusion attenuation compensation factor *C_ij_* can be approximated as follows:

(5) $$C_{ij}\left(x_{P},z_{P}\right)=\frac{1}{\sqrt{r_{iP}r_{jP}}}.$$

#### 2.2.2. Scattering Attenuation Compensation

Austenitic stainless steel welds exhibit a coarse grain structure, resulting in significant attenuation of ultrasonic signals due to strong scattering. The measured attenuation coefficient typically consists of two components: the absorption attenuation coefficient, denoted as *α_a_*, and the scattering attenuation coefficient, denoted as *α_s_*. Therefore, the attenuation coefficient can be expressed as follows:

(6) $$\alpha =\alpha_{a}+\alpha_{s}.$$

In solid media, the absorption attenuation of ultrasonic waves is relatively negligible compared to the scattering attenuation. Therefore, the measurement formula for the scattering attenuation coefficient *α_s_* can be expressed as follows:

(7) $$\alpha_{s}\approx \alpha =\frac{20\mathrm{lg}\left(A_{m}/A_{n}\right)-\delta }{2\left(n-m\right)T}$$

where *α* represents the attenuation coefficient of the material. The variables *A_m_* and *A_n_* denote the amplitudes of the mth and nth bottom surface echoes, respectively. *δ* represents the bottom surface reflection loss, which is typically assumed to be 0.5 dB. The variable *T* corresponds to the thickness of the test block.

The scattering attenuation compensation factor is denoted as *B_ij_*, which can be expressed as follows:

(8) $$B_{ij}\left(x_{P},z_{P}\right)=1+10^{\left(\frac{\alpha_{r}\left(r_{iP}+r_{jP}\right)}{20}\right)}$$

where *α_r_* represents the relative scattering attenuation coefficient of the weld, which needs to be determined through microscopic analysis. Its expression is as follows:

(9) $$\alpha_{r}=\alpha_{s-weld}-\alpha_{s-base}$$

where *α_s-weld_* denotes the scattering attenuation coefficient of the weld, while *α_s-base_* represents the scattering attenuation coefficient of the base material.

Sound field correction in TFM imaging (C-TFM) integrates the three aforementioned compensation factors to reconstruct the image, which can be expressed as follows:

(10) $$I_{C\text{-}\mathrm{TFM}}\left(x_{p},z_{p}\right)=\left|H\left(\sum_{i=1}^{N_{e}}\sum_{j=1}^{N_{e}}D_{ij}\cdot C_{ij}\cdot B_{ij}\cdot S_{ij}\left(t_{ij}\left(x_{p},z_{p}\right)\right)\right)\right|.$$

### 2.3. Vector-Coherent Imaging

Acoustic field compensation correction enhances the amplitude uniformity of TFM images, yet it can scarcely differentiate between defects and structural noise. Phase coherence imaging evaluates the phase dispersion of full matrix data. By combining the phase coherence factor with C-TFM, while incorporating phase information from the signal, noise caused by phase scattering is reduced, all while preserving amplitude uniformity. Therefore, the image SNR is improved. The Vector Coherence Factor (VCF) defines a set of unit vectors on the complex plane using the instantaneous phase. The magnitude of the vector sum quantifies phase consistency. If all phases are identical, i.e., if the signal phases are perfectly coherent, the magnitude of the vector sum approaches 1. Conversely, if the phases are uniformly distributed along the unit circle, the magnitude tends toward 0. A brief overview of the VCF calculation process is provided below [28].

First, define the complex analysis of the delayed signal, which is given by the following:

(11) $$S_{ij}\left(x,z\right)=\left|S\right|e^{i\phi_{ij}}.$$

Considering the in-phase and quadrature components of the instantaneous phase of the signal, the VCF at a given imaging point (*x*, *z*) in the imaging space can be defined as follows:

(12) VCFx,z=∑i=1Ne∑j=1NeReSijx,zSijx,z2+∑i=1Ne∑j=1NeImSijx,zSijx,z20.5

where Re represents the real part, Im denotes the imaginary part, and *N_e_* refers to the number of elements.

Using the VCF as a weighting coefficient, multiply it by the amplitude of the C-TFM image to reconstruct the acoustic field-corrected, vector-coherent TFM image, denoted as C-VCF-TFM:

(13) $$I_{C\text{-}\mathrm{VCF}-\mathrm{TFM}}\left(x,z\right)=\mathrm{VCF}\left(x,z\right)\times I_{C\text{-}\mathrm{TFM}}\left(x,z\right).$$

## 3. Result Analysis and Discussion

### 3.1. Test Blocks

**Figure 3:**
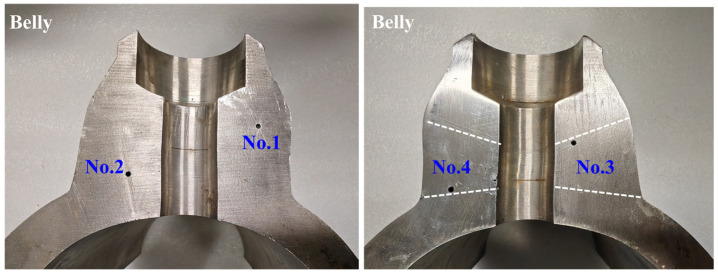

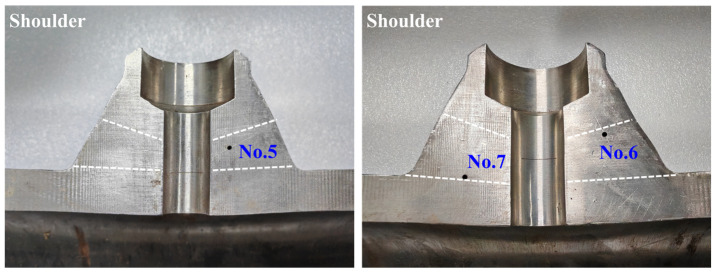
Nozzle weld test blocks with SDH defects.

Taking the weld seam of the SKETCH14 specification, a typical coarse-grained elastic anisotropic material, as an example, the main pipe has a wall thickness of 17 mm and an outer diameter of 166.5 mm, while the branch pipe has a wall thickness of 33.5 mm and an outer diameter of 84 mm. A belly test block is obtained by cutting perpendicular to the axis of the main pipe, and a shoulder test block is cut parallel to the axis of the main pipe. Seven Φ 2 side-drilled holes (SDH) are introduced at varying positions and depths within the weld seam areas of the belly and shoulder test blocks. Photographs of the test blocks and defect parameters are shown in Figure 3, where the positions of the upper and lower fusion lines are indicated by white dashed lines. The defect depth information is provided in Table 1.

**Figure 4:**
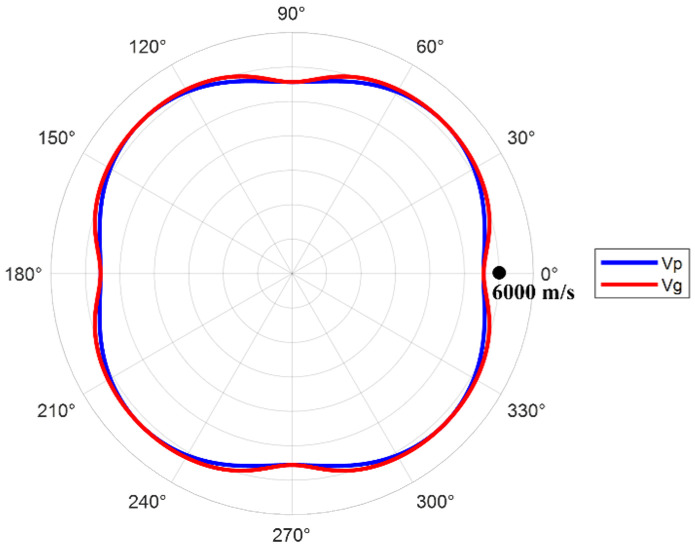
Distribution curve of ultrasonic phase/group velocity varying with propagation direction in weld material.

The material properties of the weld and the base metal area of the test blocks are shown in Table 2, including the Young’s modulus, Poisson’s ratio, density, longitudinal wave sound velocity, and attenuation coefficient. The distribution curve of the phase/group velocity of ultrasonic waves varying with the propagation direction in the weld material is shown in Figure 4. It can be observed that there is obvious anisotropy in the weld, which affects the propagation speed of ultrasonic waves inside it. The instrument used in the phased array ultrasound experiment is a 128-channel ultrasound board, equipped with a 32-element phased array probe that has a center frequency of 5 MHz and an element center distance of 0.5 mm. The detection method employed is the contact method, and full matrix data for the seven defects were collected separately.

### 3.2. Simulation Analysis

The nozzle weld seam of austenitic stainless steel exhibits a coarse grain structure and strong anisotropy. Structural variations in different regions lead to varying degrees of impact on ultrasonic refraction, scattering, and other phenomena, resulting in non-uniformity in TFM imaging and a reduction in image quality. This study employs EBSD analysis and modeling simulations to examine the effects of the base metal, heat-affected zone, and weld area on ultrasonic signal scattering, and to optimize the selection of scattering attenuation compensation factors for different regions.

**Figure 5:**
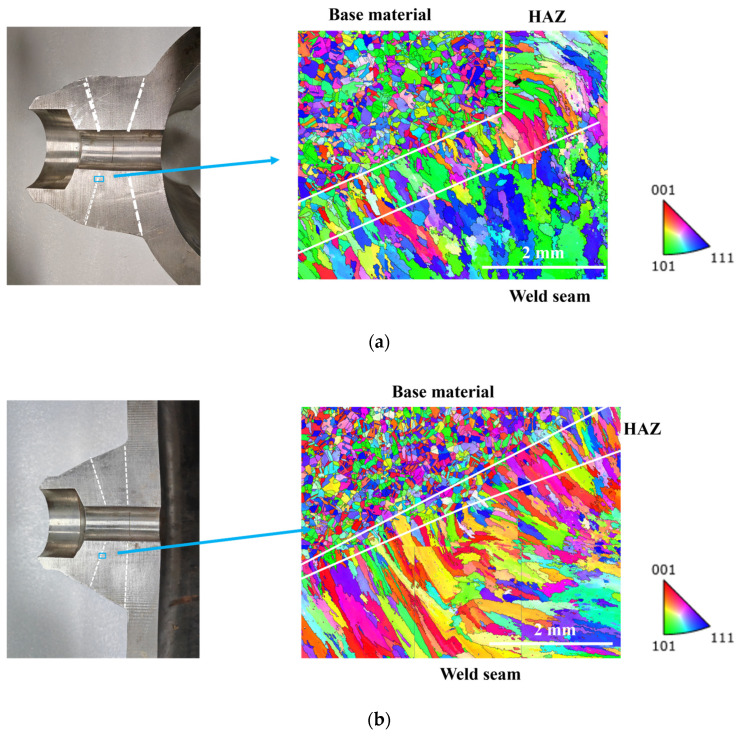
EBSD map of weld seams of austenitic stainless steel: (**a**) belly; (**b**) shoulder.

Along the detection imaging cross-section, samples with dimensions of 16 × 12 × 8 mm were extracted from the weld zones of the belly and shoulder test blocks. Using the EBSD probe of a high-resolution field emission scanning electron microscope, multiple diffraction patterns in the weld and base metal regions were captured. A map (with a size of 5 × 4 mm) formed by stitching together six typical regions selected from each block is shown in Figure 5. From the figure, it can be observed that clear boundaries exist between the weld seam, heat-affected zone, and base metal area. The base metal region consists of uniformly equiaxed fine grains, with an average equivalent circle diameter of 38.92 μm. The heat-affected zone contains elongated columnar crystals aligned with the cooling direction of the weld seam, with grain lengths ranging from 89.44 to 1128.55 μm and widths ranging from 90.24 to 145.78 μm. The weld area is characterized by coarse grains with noticeable directional alignment, and the maximum equivalent circle diameter of the grains can reach 1545.62 μm.

**Figure 6:**
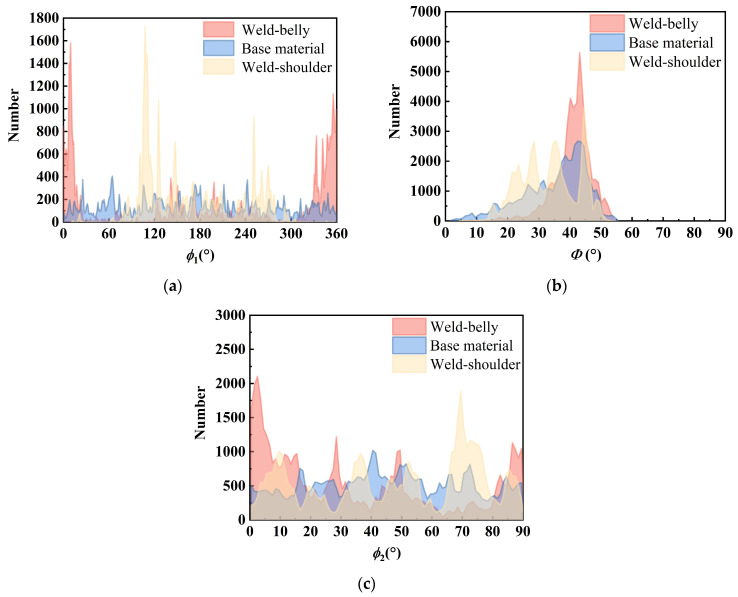
Comparison of Euler angle distributions of grains in weld seam and base metal area: (**a**) *ϕ*_1_; (**b**) Φ; (**c**) *ϕ*_2_.

The grain orientations of the weld seam and base metal area were extracted, and the distributions of their Euler angles are shown in Figure 6. From the figure, it can be observed that the Euler angle distributions *ϕ*_1_ and *ϕ*_2_ of the base material are relatively uniform, with a peak in Φ, primarily due to the orientation induced by pipeline rolling. The three Euler angles of the belly and shoulder welds exhibit one or more peaks. In addition to the rolling direction, a preferred orientation of grain growth is also observed due to welding cooling, resulting in significant anisotropy.

**Figure 7:**
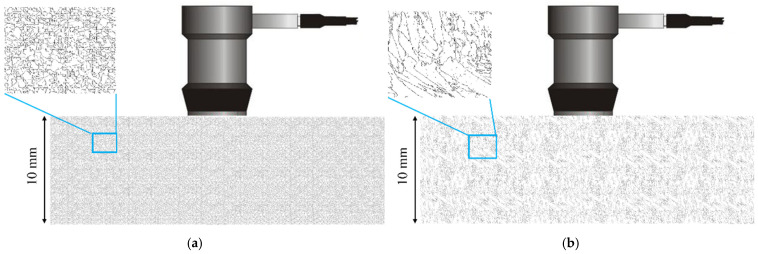
Attenuation measurement simulation model setup: (**a**) weld seam; (**b**) base material.

The cubic elastic constants in the weld sample coordinate system are C_11_ = 241 GPa, C_12_ = 138 GPa, and C_44_ = 112 GPa, respectively. Based on the grain distribution results in different regions of the EBSD map, the elastic constants of different orientation regions are obtained by the Euler angle method. An ultrasonic simulation model of austenitic stainless steel welds was established by using the finite difference method [29]. The two sides were set as absorption boundaries and the bottom surface as a rigid boundary. To analyze the scattering effects of ultrasound signals in various regions, separate models for the weld seam and base metal, each with a thickness of 10 mm, were constructed (Figure 7). An ultrasound probe with a center frequency of 5 MHz was employed to acquire ultrasound signals, and the attenuation of ultrasound waves with propagation distance in both the weld seam and base metal regions was analyzed.

**Figure 8:**
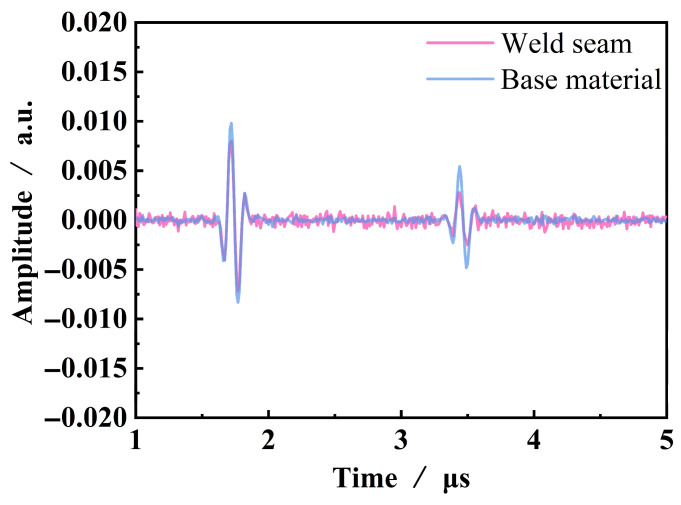
Amplitude attenuation of ultrasonic testing in different regions.

The primary and secondary bottom echo signals of the base metal and weld areas are shown in Figure 8. It can be observed that the two bottom echoes in the base material are significantly higher than those in the weld area, and the amplitude of the second bottom echo decreases less than that of the first bottom echo. Compared to the base material, there is noticeable noise in the ultrasonic signals collected from the weld area between the first and second bottom echoes. This is primarily due to the small grain size and uniform orientation distribution in the base material, which results in a weak scattering effect of ultrasonic waves by the grains, and the influence of propagation distance on energy attenuation is relatively small. In contrast, the weld area exhibits a coarse grain structure, which has a strong scattering effect on ultrasonic waves, leading to significant scattering attenuation. Consequently, this results in strong noise and weak bottom echoes in the signal. The range of scattering attenuation coefficients for the base metal and weld areas is 0.17–0.26 dB/mm and 0.33–0.45 dB/mm, respectively. Therefore, the relative scattering attenuation coefficient *α_r_* is 0.16–0.28 dB/mm. Taking into account the compensation effect, an average value of 0.22 dB/mm is used as the relative scattering attenuation coefficient.

**Figure 9:**
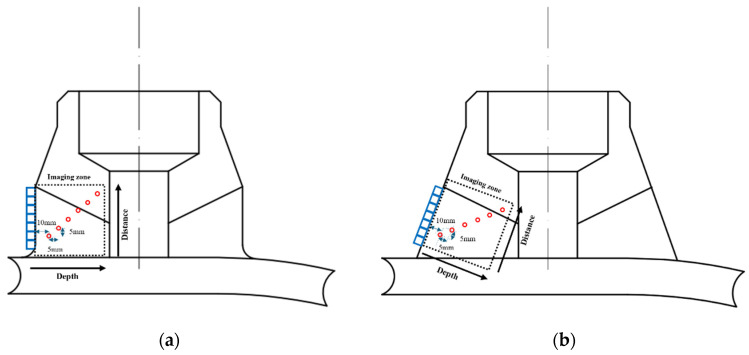
Defect setup for imaging comparison model: (**a**) belly; (**b**) shoulder.

To compare the imaging effects of different sound field compensation methods before and after correction, six 2 mm diameter SDHs with varying depths were processed in the nozzle weld belly and shoulder ultrasound simulation model, as shown in Figure 9. The defect depth range was set from 10 mm to 35 mm, and the horizontal distance between adjacent defects was 5 mm. This setup was designed to evaluate the compensation effects of different sound field compensation methods in both the horizontal and vertical directions.

**Figure 10:**
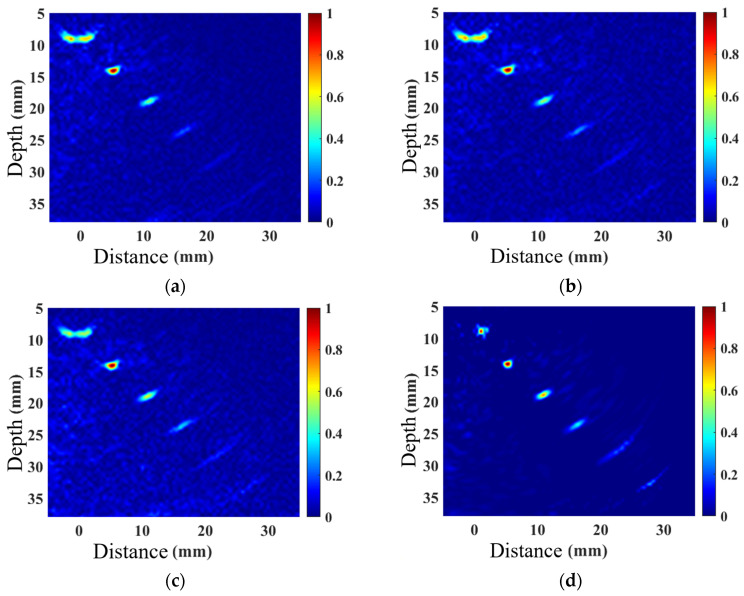
Comparison of weld belly simulation imaging: (**a**) TFM; (**b**) introduction of directional function correction for sound field; (**c**) incorporation of diffusion attenuation; (**d**) C-VCF-TFM.

**Figure 11:**
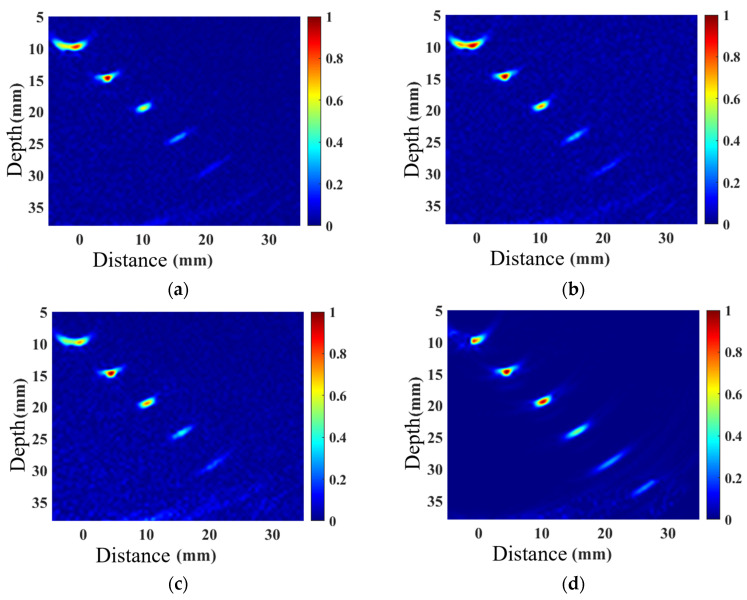
Comparison of weld shoulder simulation imaging: (**a**) TFM; (**b**) introduction of directional function correction for sound field; (**c**) incorporation of diffusion attenuation; (**d**) C-VCF-TFM.

**Figure 12:**
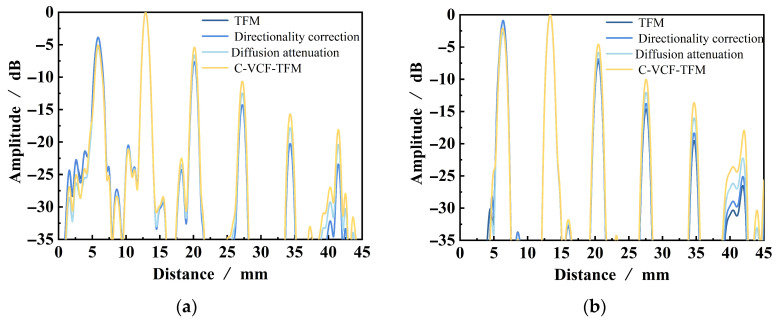
Comparison of defect amplitude between different imaging methods: (**a**) belly; (**b**) shoulder.

A linear array probe with a center frequency of 5 MHz and 32 elements was used to perform TFM imaging on the belly and shoulder defects, as well as acoustic field-corrected vector-coherent TFM imaging using different correction functions. The results are shown in Figure 10 and Figure 11. TFM (Figure 10 and Figure 11a) can identify SDH defects at depths ranging from 10 mm to 25 mm, while defects at depths of 30 mm and above are difficult to detect. The imaging effect of applying only the directional function of the sound field (Figure 10 and Figure 11b) shows minimal improvement, with the amplitude of the SDH defect at a depth of 25 mm slightly increasing. After incorporating diffusion attenuation into the sound field correction function, the deepest SDH defect, measuring 30 mm, can be identified in the image (Figure 10 and Figure 11c), but at this point, significant background noise is introduced, affecting image quality. The C-VCF-TFM image provides imaging of all six SDH defects (Figure 10 and Figure 11d), with enhanced defect amplitudes at depths ranging from 20 mm to 30 mm and no divergence. The comparison results of defect amplitudes using different imaging methods are shown in Figure 12. As can be observed from the figure, the defect amplitudes in C-VCF-TFM have been improved to varying extents, and there is no significant artifact interference around the defect amplitudes, which enhances the defect detection capability. Based on the comprehensive imaging results, it can be concluded that, compared to TFM imaging and the other two types of acoustic field correction imaging, the defect SNR of C-VCF-TFM imaging has increased by 0.54 dB to 6.67 dB, effectively suppressing the attenuation caused by coarse grains and the non-uniformity of acoustic field distribution, thereby expanding the effective detection range of weld TFM imaging.

### 3.3. Experimental Result

**Figure 13:**
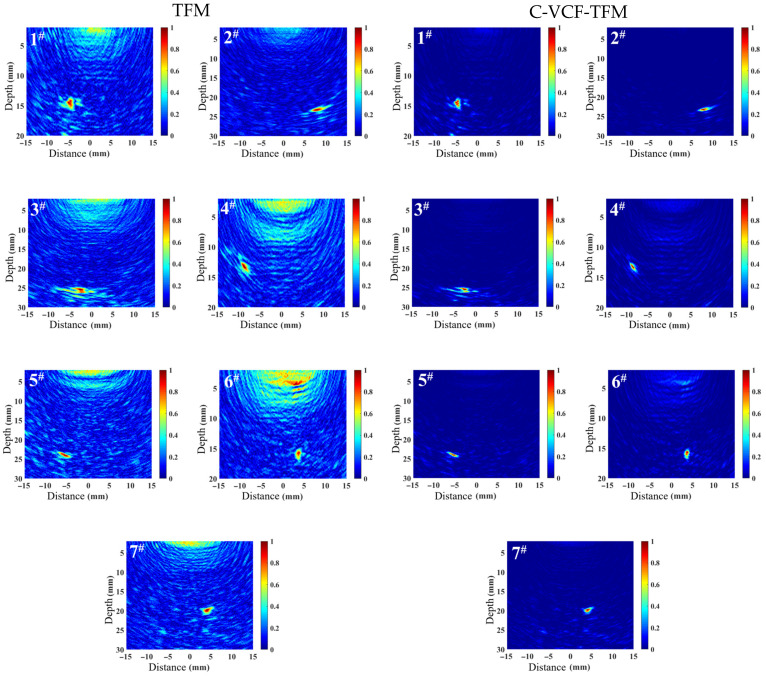
Imaging comparison of typical defects in weld seams.

The TFM imaging and C-VCF-TFM imaging results for some typical defects in the welds, respectively labeled as 1^#^–7^#^, are shown in Figure 13. From the figure, it can be observed that the background noise in the TFM image is relatively high, with artifacts generated in the near-surface area due to the proximity of the ultrasonic excitation position of the array element. Significant shadowing is observed in the defects, accompanied by strong structural noise in the vicinity, which affects the quality of the TFM image. In contrast, the background noise in the C-VCF-TFM image is suppressed, the defect shadowing is reduced, and the amplitude of near-surface artifacts is diminished.

The testing SNRs and defect depth localization errors of TFM imaging and C-VCF-TFM imaging were compared. To this end, the SNR was calculated by Equation (14) [30]:

(14) $$\mathrm{SNR}=20\mathrm{lg}\left(\frac{I_{\mathrm{signal}}}{I_{\mathrm{noise}}}\right)$$

where *I*_signal_ and *I*_noise_ are, respectively, the amplitude of the defect image and the average amplitude of the nearby noise artifact.

**Figure 14:**
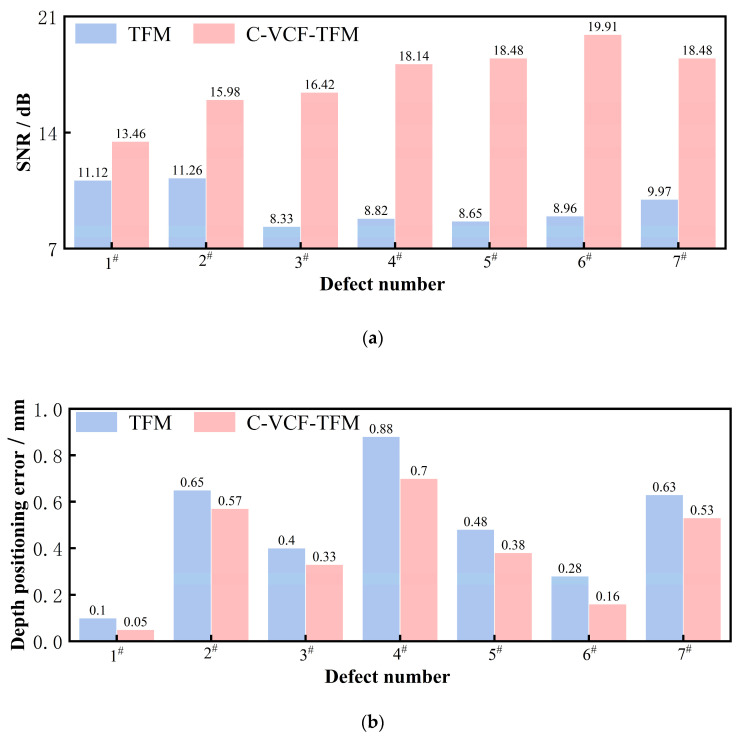
Comparison of imaging performance of weld seam test blocks in austenitic stainless steel: (**a**) SNR; (**b**) depth positioning error.

The calculation method for the defect depth location error is as follows:

(15) $$e_{d}=|Z_{1}-Z_{2}|$$

where *Z*_1_ represents the depth at which the maximum-size pixel *P*_1_ (*x*,*z*) of the defect image is located, and *Z*_2_ is the actual depth designed for the defect. The results are presented in Figure 14. Compared to TFM imaging, the SNR of C-VCF-TFM imaging increased by 2.34–10.95 dB, while the positioning error decreased from 0.1 mm–0.88 mm to 0.05 mm–0.70 mm. The acoustic field uniformity of the TFM images is inadequate. Near the surface, due to the proximity of the phased array ultrasonic probe emitting ultrasonic waves, the amplitude of the acoustic field is strong. As the depth and propagation distance increase, the ultrasonic waves undergo diffusion attenuation, scattering, refraction, and other effects, leading to a gradual decrease in energy. Consequently, the energy distribution within the imaging space becomes uneven. However, TFM imaging assumes uniform energy distribution at every point in the space, resulting in the formation of artifacts due to uneven energy amplitude, such as artifacts caused by the superposition of array element emissions near the surface. In the weld of austenitic stainless steel, the coarse grain structure causes more severe scattering and refraction of ultrasonic waves. In ultrasonic images, this results in weak reflectivity, further elevating the background noise level. In TFM imaging, this manifests as a false image, and it is challenging to effectively suppress the noise caused by the coarse grain structure using acoustic field correction methods alone. C-VCF-TFM imaging compensates for both the diffusion and scattering attenuation of ultrasonic waves. Based on the directional function of the acoustic field, the energy distribution within the imaging space is adjusted. By utilizing phase information, defects and structural noise are distinguished based on the phase coherence of each point in the imaging space. The energy amplitude of non-scattering regions is adaptively suppressed. With the additional acoustic field correction compensation, the level of structural noise is further reduced, significantly improving the imaging SNR, mitigating the impact of acoustic field non-uniformity on image quality, reducing artifacts near defects, and enhancing detection accuracy.

## 4. Conclusions

1. This article proposes a sound field correction vector-coherent total focusing imaging method that incorporates material properties. Based on the scattering variations caused by differences in the microstructure of the austenitic stainless steel weld base material and weld zone, the scattering attenuation compensation factor Bij is optimized and selected to construct a sound field correction function, reducing the impact of sound field non-uniformity on image quality. By utilizing phase information, the phase coherence of each point in the imaging space is evaluated to further suppress structural noise. This method improves the signal-to-noise ratio, suppresses artifacts, and corrects defect imaging positions, thereby achieving high-precision detection.
2. Ultrasonic experiments were conducted on seven transverse through-hole defects at various positions on the weld belly and shoulder test blocks. The results indicate that, compared to fully focused imaging, the detection signal-to-noise ratio improvement range for the acoustic field correction vector-coherent fully focused imaging method is 2.34 dB to 10.95 dB, while the positioning error decreases from 0.1–0.88 mm to 0.05–0.70 mm.

## Figures and Tables

**Table 1:** Nozzle weld defect depth configuration.

No.	1	2	3	4	5	6	7
Defect depth (mm)	14.4	22.6	26.0	12.5	24.5	15.5	20.5

**Table 2:** Material properties of austenitic stainless steel welds.

	Base Material	Weld Seam
Young’s Modulus (GPa)	194	190
Poisson’s ratio	0.285~0.291	
Density (g/cm^3^)	7.93	
Longitudinal wave velocity (m/s)	5769.2~5836.3	5826.5~5860.3
Attenuation coefficient (dB/mm)	0.11~0.12	0.20~0.21

## Data Availability

The data are contained within the article.

## Abbreviations

The following abbreviations are used in this manuscript:

TFM	Total Focusing Method
SNR	Signal-to-noise ratio
VCF	Vector Coherence Factor
SDH	Side-drilled hole
